# Observation of Spin Splitting in Room‐Temperature Metallic Antiferromagnet CrSb

**DOI:** 10.1002/advs.202406529

**Published:** 2024-09-20

**Authors:** Meng Zeng, Ming‐Yuan Zhu, Yu‐Peng Zhu, Xiang‐Rui Liu, Xiao‐Ming Ma, Yu‐Jie Hao, Pengfei Liu, Gexing Qu, Yichen Yang, Zhicheng Jiang, Kohei Yamagami, Masashi Arita, Xiaoqian Zhang, Tian‐Hao Shao, Yue Dai, Kenya Shimada, Zhengtai Liu, Mao Ye, Yaobo Huang, Qihang Liu, Chang Liu

**Affiliations:** ^1^ Department of Physics and Shenzhen Institute for Quantum Science and Engineering (SIQSE) Southern University of Science and Technology (SUSTech) Shenzhen Guangdong 518055 China; ^2^ Beijing National Laboratory for Condensed Matter Physics and Institute of Physics Chinese Academy of Sciences Beijing 100190 China; ^3^ State Key Laboratory of Functional Materials for Informatics Shanghai Institute of Microsystem and Information Technology Chinese Academy of Sciences Shanghai 200050 China; ^4^ National Synchrotron Radiation Laboratory and School of Nuclear Science and Technology University of Science and Technology of China Hefei Anhui 230026 China; ^5^ Japan Synchrotron Radiation Research Institute (JASRI) Sayo Hyogo 679‐5198 Japan; ^6^ Hiroshima Synchrotron Radiation Centre Hiroshima University Higashi‐Hiroshima Hiroshima 739‐0046 Japan; ^7^ Key Laboratory of Quantum Materials and Devices of Ministry of Education School of Physics Southeast University Nanjing Jiangsu 211189 China; ^8^ Shanghai Synchrotron Radiation Facility Shanghai Advanced Research Institute Chinese Academy of Sciences Shanghai 201204 China

**Keywords:** angle‐resolved photoemission spectroscopy, density functional theory calculations, spin splitting antiferromagnet, spintronics, unconventional antiferromagnet

## Abstract

Recently, unconventional antiferromagnets that enable the spin splitting (SS) of electronic states have been theoretically proposed and experimentally realized, where the magnetic sublattices containing moments pointing at different directions are connected by a novel set of symmetries. Such SS is substantial, *k*‐dependent, and independent of the spin–orbit coupling (SOC) strength, making these magnets promising materials for antiferromagnetic spintronics. Here, combined with angle‐resolved photoemission spectroscopy (ARPES) and density functional theory (DFT) calculations, a systematic study on CrSb, a metallic spin‐split antiferromagnet candidate with Néel temperature *T_N_* = 703 K, is conducted. The data reveal the electronic structure of CrSb along both out‐of‐plane and in‐plane momentum directions, rendering an anisotropic *k*‐dependent SS that agrees well with the calculational results. The magnitude of such SS reaches up to at least 0.8 eV at non‐high‐symmetry momentum points, which is significantly higher than the largest known SOC‐induced SS. This compound expands the choice of materials in the field of antiferromagnetic spintronics and is likely to stimulate subsequent investigations of high‐efficiency spintronic devices that are functional at room temperature.

## Graphical abstract

A substantial antiferromagnetism‐induced momentum‐dependent band splitting is observed in room‐temperature metallic antiferromagnet CrSb. Using high‐resolution angle‐resolved photoemission spectroscopy measurements, clear evolution of band splitting along both out‐of‐plane and in‐plane directions is realized. This work provides spectroscopic evidence for CrSb as a spin‐split antiferromagnet and showcases its potential for applications in the evolving landscape of antiferromagnetic spintronics.

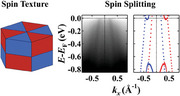

## 1 Introduction

In the framework of group theory, nonmagnetic materials without spin–orbit coupling (SOC), nonmagnetic materials with SOC, and magnetic materials with SOC can be fully characterized by space groups, double space groups, and magnetic double space groups, respectively. However, there are antiferromagnetic (AFM) materials in which SOC can be theoretically set to zero as a starting point to examine the essential physical properties. The symmetry of these magnets is rarely explored. These materials possess a crucial property that the spin degree of freedom is partially decoupled from the orbital part,^[^ ^1^, ^2^, ^3^, ^4^, ^5^, ^6^, ^7^, ^8^, ^9^ ^]^ resulting in a substantial and momentum‐dependent spin splitting (SS) whose energy scale is often much larger than that of the SSs caused by SOC. The spin texture of such band splitting remains scarcely measured experimentally.^[^ ^10^, ^11^, ^12^, ^13^, ^14^, ^15^ ^]^ To establish the theoretical underpinnings of such innovative antiferromagnets, Hayami et al. proposed that the anisotropic kinetic motion of electrons gives rise to an effective SOC, resulting in an anisotropic SS.^[^ ^10^, ^11^ ^]^ Yuan et al. proposed several prototypes of SS and predicted the presence of AFM‐induced spin separation.^[^ ^13^, ^14^, ^15^ ^]^ A number of research groups introduced the concept of the spin group^[^ ^1^, ^2^, ^3^, ^4^, ^5^, ^6^, ^7^, ^8^, ^9^ ^]^ to describe the electronic structure of such materials and predicted a series of associated physical phenomena, such as the weak SOC *Z*_2_ topological phase,^[^ ^4^ ^]^ the chiral Dirac‐like fermions,^[^ ^16^, ^17^ ^]^ the *C*‐pair spin valley locking,^[^ ^18^ ^]^ the nonrelativistic spin Hall effect,^[^ ^19^, ^20^ ^]^ the spin splitter torque,^[^ ^21^, ^22^ ^]^ the nonrelativistic Edelstein effect,^[^ ^23^ ^]^ and the anomalous Hall effect.^[^ ^24^, ^25^ ^]^ Specifically, the term “altermagnet” is introduced to designate *collinear* antiferromagnets that possess such AFM‐induced SS.^[^ ^26^, ^27^, ^28^ ^]^

In recent years, unconventional antiferromagnetism has gained significant traction and emerged as a promising research field. Fruitful findings have been unveiled, such as the anomalous Hall effects in RuO_2_^[^ ^24^ ^]^ and MnTe,^[^ ^29^ ^]^ and the plaid‐like spin splitting in MnTe_2_.^[^ ^30^ ^]^ However, certain constraints exist within the materials mentioned above. For example, despite substantial progress in understanding the unconventional antiferromagnetic nature of RuO_2_,^[^ ^21^, ^22^, ^24^, ^31^ ^]^ there is still controversy about its ground state magnetism.^[^ ^32^, ^33^, ^34^ ^]^ In addition, MnTe and MnTe_2_, both of which are semiconductors with bandgaps,^[^ ^30^, ^35^, ^36^ ^]^ exhibit suboptimal conductivity compared to metals, limiting their potential applications in spintronics.

In this article, we focus on CrSb, a candidate of unconventional antiferromagnet with Néel temperature significantly surpassing the room temperature (*T*_*N*_ = 703 K for the bulk^[^ ^37^ ^]^). Compared to MnTe, CrSb is an out‐of‐plane *A*‐type AFM metal, offering better conductivity and magnetic storage density. Compared to RuO_2_, CrSb demonstrates higher phase transition temperature, larger effective magnetic moment and more pronounced anisotropic SS, resulting in higher spin torque conductivity and evident anomalous Hall effect.^[^ ^21^, ^24^, ^38^ ^]^ Hence, CrSb stands out as an ideal material for spintronic application. Previous investigations into the electronic structure of CrSb have been conducted on thin films using soft X‐ray ARPES.^[^ ^39^ ^]^ Here, we employ high‐resolution vacuum ultraviolet (VUV) and soft X‐ray ARPES to probe the SS in CrSb. We compared the magnitude of band splitting at different photon energies and in‐plane momenta, clearly observing the evolution of splitting along both out‐of‐plane and in‐plane directions. Such anisotropic band splitting matches the theoretical prediction based on the unique symmetry of this unconventional spin‐split antiferromagnet. Even without spin‐ARPES results, the splitting behavior is found to be distinct from the textures of traditional SOC‐induced spin splitting. Our experimental results provide spectroscopic evidence for CrSb as a spin‐split antiferromagnet, and showcase its potential for applications in the evolving landscape of antiferromagnetic spintronics.

## 2 Results and Discussion

**Figure 1:**
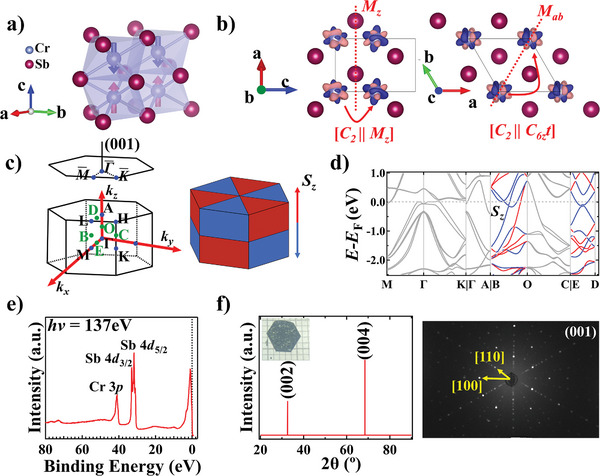
High‐Néel‐temperature metallic spin‐split antiferromagnet CrSb. a) Crystal structure of CrSb along with magnetic moments. Red/blue arrows indicate the spin‐up and ‐down orientations of the Cr atom. b) Spin‐polarized electronic density distribution of CrSb. Black lines indicate the unit cell. Red dash lines indicate the mirror planes. Transpose symmetries [*C_2_* || *M_z_*] and [*C_2_* || *C_6z_t*] are shown. c) Left: the 3D and surface‐projected Brillouin zone (BZ) of CrSb. Blue dots represent high symmetry points; green dots represent the midpoint between two high symmetry points. Right: Schematic for the sign of *S_z_* polarization in the 3D BZ. d) DFT‐calculated spin‐resolved bands with SOC. There is no spin polarization within the *ΓMK* and *ΓKHA* plane, whereas a high level of spin polarization is seen in the *OBC* and *ΓMLA* plane. e) Core‐level photoemission spectra taken on an in situ cleaved sample at *hν* = 137 eV. f) Single crystal XRD results and representative Laue X‐ray diffraction pattern of the (001) surface of CrSb. Yellow arrows in the Laue pattern show the orientation indexes of [100] and [110]. Inset: a hexagonally shaped CrSb single crystal with exposed (001) plane against a millimeter gird.

The crystal structure of CrSb is shown in **Figure** **1**a. It crystallizes in the NiAs‐type crystal structure with the space group *P*6_3_/*mmc* (#194). The Cr atom is surrounded by six Sb atoms, forming an octahedron with twofold rotational symmetry. Below the Néel temperature, it exhibits an out‐of‐plane *A*‐type AFM ground state where the spins align ferromagnetically in‐plane and antiferromagnetically between adjacent layers. Figure 1b displays the spin‐polarized electronic density distribution map of CrSb. The electronic density between two Cr atoms cannot be linked through inversion or translation. Instead, the sublattices are linked by mirror symmetry *M_z_* or rotation symmetry *C*_6_ *_z_*, corresponding to the spin group element [*C*_2_ || *M_z_*] or [*C*_2_ || *C*_6_ *_z_t*] for adjacent Cr atoms along the out‐of‐plane or in‐plane direction (R*_i_* and R*_j_* in [R*_i_* || R*_j_*] denote operations in the spin and lattice space, respectively). Under these symmetries, the Kramers degeneracy cannot be maintained within the whole Brillouin zone (BZ), yielding the nonrelativistic SS.

The 3D and surface‐projected BZ of CrSb, together with the sign of the *c*‐direction spin polarization (*S_z_*) in the 3D BZ, are shown in Figure 1c (the blue/red triangular prisms represent the negative/positive spin components). *M_z_* corresponds to one horizontal mirror plane, enforcing that the bands are degenerate at the *GMK* and the *ALH* planes; *C*_6_ *_z_* combining with *M_ab_* corresponds to three vertical mirror planes, indicating that bands are degenerate within the *ΓKHA* plane. The sign of *S_z_* is antisymmetric about the mirror planes, forming an alternative pattern in the 3D BZ.

The DFT‐calculated *E*‐*k* dispersion with SOC are shown in Figure 1d. The high‐symmetry momentum directions *M‐Γ‐K* and *Γ‐A* manifest no AFM‐induced SS, while significant AFM‐induced SS is seen to exist along non‐high‐symmetry momentum directions *B‐O* and *E‐D*. The magnitude of SS can reach up to 1.1 eV. Though antimony is expected to have a pronounced SOC effect on the electronic structure, the magnitude of SOC‐induced SS in high‐symmetry points and the band intersections are no more than ≈0.1 eV, far below the total SS magnitude (Section SIV, Supporting Information). Therefore, the substantial SS in CrSb is induced overwhelmingly by the AFM order.

Single crystals of CrSb were grown by the chemical vapor transport (CVT) method. The core‐level photoemission spectrum reveals the occupied 4*d* orbitals of the Sb atoms and the 3*p* orbital of the Cr atoms (Figure 1e, see also Section SVII, Supporting Information). In Figure 1f, the X‐ray diffraction (XRD) results reveal the (00*l*) peaks of CrSb, consistent with the Laue X‐ray diffraction pattern. These results are indicative of the high quality of the crystals (see also Sections SI–SIII, Supporting Information).

**Figure 2:**
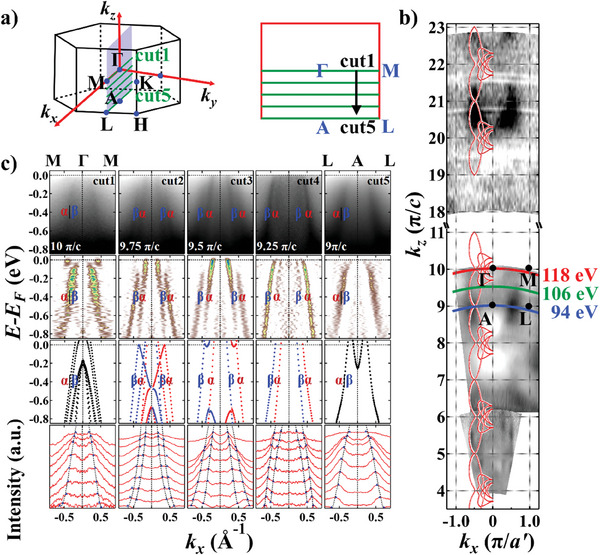
Evolution of the band splitting at different out‐of‐plane momentum positions. a) 3D BZ of CrSb with a 2D cross‐section showing the *ΓMLA* plane. The green lines represent measurement positions for cuts 1–5. b) ARPES *k_z_*–*k_x_* CECs at binding energies *E*_B_ = 0.6 eV, *a*′ = $\left(\sqrt{3}/2\right)$ *a*. The inner potential was set to be *V*_0_ = 12.8 eV. High symmetry points and *k_z_*–*k_x_* curves corresponding to three typical photon energies are labeled. The appended red curves represent the DFT calculational results. c) From top to bottom: ARPES band dispersion parallel to $\bar{\Gamma }$‐$\bar{M}$ with an equal *k_z_* offset of 0.25 π/*c* between each; corresponding second derivative analysis along the EDCs; DFT‐calculated bulk bands (all calculated bands are shifted up by 0.15 eV to account for the charged defects in real crystals), where red and blue correspond to out‐of‐plane spin‐up and ‐down polarization, respectively; MDCs with peak fitting results. MDCs are drawn with an energy offset of 0.1 eV from *E*_B_ = 0 eV to *E*_B_ = 0.8 eV. Blue triangles denote the MDC peaks, and the black dashes represent the fitting curves.

As mentioned above, the bands of CrSb are degenerated at *k_z_* = *n*π/*c* (*n* = 0, 1, 2, 3, …) and split at positions where *k_z_* ≠ *n*π/*c* and (*k_x_*, *k_y_*) are not within the *ΓKHA* planes. Therefore, the periodic evolution of SS can be revealed within the *ΓMLA* plane and the *OBC* plane. To illustrate the anisotropic SS within the *ΓMLA* plane, we carried out systematic *hν*‐dependent ARPES measurements with in‐plane momentum aligned along $\bar{\Gamma }$‐$\bar{M}$ (**Figure** **2**). The *k_z_*‐*k_x_* constant energy contours (CECs) demonstrate clear periodic out‐of‐plane dispersion (Figure 2b). Through comparison with computational results, the inner potential *V*_0_ was determined to be 12.8 eV (Section SV, Supporting Information). By analyzing the data, we also found that the bands resolved under 94–118 eV photons are mostly bulk states, while those under 12–37 eV photons are mostly surface states (Section SIX, Supporting Information). Therefore, we used the data taken with 94 eV < *hν* < 118 eV for analysis of the bulk bands. We have not seen obviously different band structures on different spatial regions of the samples. Therefore, we assume that the cleavage plane of our sample is a mixture of Sb‐ and Cr‐terminated surfaces. The *E*_B_‐*k_x_* dispersion of CrSb at different *k_z_*’s (designated as cuts 1–5 in Figure 2a) are shown in Figure 2c. The four rows in Figure 2c correspond to the raw data, the second derivative analysis along the energy distribution curves (EDCs), the DFT‐calculated bulk bands with SOC, and the momentum distribution curves (MDCs) along $\bar{M}$‐$\bar{\Gamma }$‐$\bar{M}$. Within these ARPES spectra, we identify two bands *α* and *β*. Even with a rough examination of the raw data in the first row, we can clearly observe that these bands are degenerate at the bulk *Γ* and *A* points (*Γ*: *hν* = 118 eV, *k_z_* = 10 π/*c; A*: *hν* = 94 eV, *k_z_* = 9 π/*c*), while split into the *α* and *β* bands away from *Γ* and *A*. At the center of *Γ‐A* (*hν* = 106 eV, *k_z_* = 9.5 π/*c*), the SS is maximal. To better visualize the SS, we perform the second derivative analysis along the EDCs and fit peaks from the raw MDCs, as shown in the second and the fourth row, respectively. Both analyses demonstrate good agreement between our experimental results and theoretical calculations (the third row in Figure 2c), endorsing the observation of giant anisotropic SS along the out‐of‐plane direction. The same trend of SS is also resolved in our soft‐X‐ray ARPES data, further confirming the AFM‐induced band splitting (Section SVIII, Supporting Information). In cut 3, where the maximum SS parallel to $\bar{\Gamma }$‐$\bar{M}$ occurs, the energy scale of it is ≈0.8 eV at *k_x_* = ± 0.439 Å^−1^, significantly higher than the largest known giant SOC‐induced SS in BiTeI (≈0.2 eV)^[^ ^40^ ^]^ and GeTe (≈0.2 eV).^[^ ^41^ ^]^ Some regions in the second derivative diagram show additional spectral weight, which may result from a combination of the *k_z_* broadening effect that reflects bands from neighboring *k_z_*’s and the existence of surface states.

**Figure 3:**
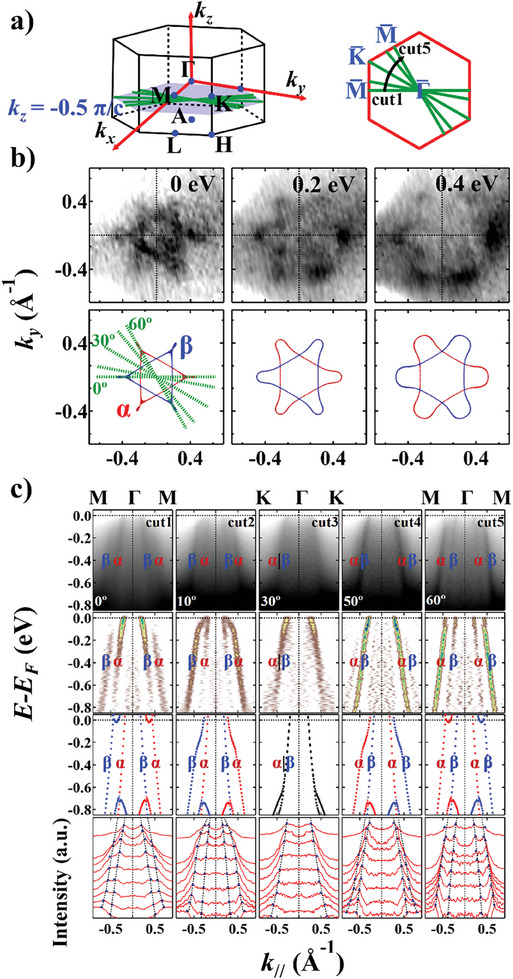
Evolution of the band splitting at different in‐plane directions. a) 3D BZ of CrSb with a 2D cross‐section showing the *OBC* plane (*hν* = 106 eV, *k_z_* = −0.5 π/*c*). Green lines represent the measurement directions of cuts 1–5 in Panel (c). b) ARPES CECs at *E*_B_ = 0, 0.2, and 0.4 eV with incident *hn* = 106 eV, and DFT calculated results. The *k_x_* direction is aligned along $\bar{\Gamma }$‐$\bar{M}$. c) From top to bottom: ARPES band dispersion along cuts 1–5; corresponding second derivative analysis along the EDCs; DFT‐calculated bulk bands along cuts 1–5; MDCs with peak fitting results. MDCs are drawn with an energy offset of 0.1 eV from *E*_B_ = 0 eV to *E*_B_ = 0.8 eV. Blue triangles denote the MDC peaks, and the black dashes represent the fitting curves.

To illustrate the anisotropic SS within the *OBC* plane, in **Figure** **3** we carried out ARPES measurements with *hν* = 106 eV [*k_z_* = 9.5 π/*c* ≡ −0.5 π/*c*]. The CECs at *E*_B_ = 0, 0.2, and 0.4 eV are shown in Figure 3b, where we see that the two triangular bands *α* and *β* form a hexagram‐like pattern resembling the star of David. As the binding energy increases, the star gradually enlarges, exhibiting the characteristics of hole bands. From the calculational results shown in the bottom panel of Figure 3b, the *α* and *β* bands possess different spin polarizations, revealing that the SS is also anisotropic within the (001) plane. To illustrate the evolution of in‐plane SS more clearly, we perform several *E*_B_‐*k*_//_ cuts along different in‐plane directions (Figure 3c). The azimuth angles of cuts 1–5 with respect to $\bar{\Gamma }$‐$\bar{M}$ are 0°, 10°, 30°, 50°, and 60°, respectively. We observe that when the cut rotates from $\bar{\Gamma }$‐$\bar{M}$ (cut 1) to $\bar{\Gamma }$‐$\bar{K}$ (cut 3) and to another $\bar{\Gamma }$‐$\bar{M}$ (cut 5), the *α* and *β* bands move closer, merges into one (along $\bar{\Gamma }$‐$\bar{K}$), and split again into the two bands. Such periodic variation conforms to the symmetry operation [*C*_2_ || *C*_6_ *_z_t*] of an SS antiferromagnet defined by the spin group shown in Figure 1b.

**Figure 4:**
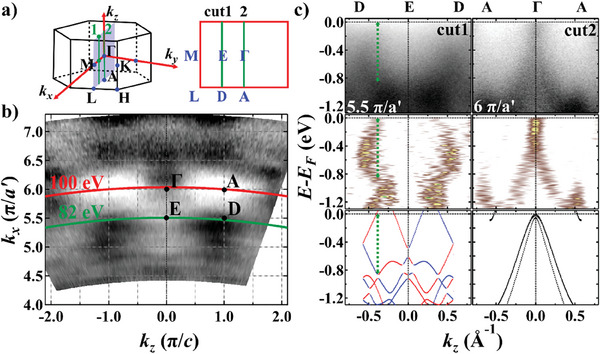
Band splitting along non‐high‐symmetry momentum direction *E–D*. a) 3D BZ of CrSb with a 2D cross‐section showing the *ΓMLA* plane. Green lines represent the measurement directions of cuts 1–2 in Panel (c). b) ARPES CEC at *E*_B_ = 0.6 eV. The inner potential was set as *V*_0_ = 11.8 eV. High symmetry points and *k_x_*–*k_z_* curves correspond to two typical photon energies are labeled. c) From top to bottom: ARPES band dispersion along cuts 1–2 with incident *hν* = 82 eV (*k_z_* = 5.5 π/*a*′) and 100 eV (*k_z_* = 6 π/*a*′), respectively; corresponding second derivative analysis along the EDCs; DFT‐calculated bulk bands (all calculated bands are shifted up by 0.31 eV to account for the charged defects in real crystals), The green dashed line mark the biggest‐seen energy separation of the split bands (≈0.8 eV).

To resolve the giant SS within the *ΓMLA* plane, we used the self‐flux method to grow another set of CrSb single crystals that terminates along the (100) plane. In **Figure** **4** we carried out systematic *hν*‐dependent ARPES measurements with in‐plane momentum aligned along $\bar{\Gamma }$‐$\bar{A}$ to pinpoint the bulk high‐symmetry points (see also Section SVI, Supporting Information). The *k_x_*‐*k_z_* CEC at *E*_B_ = 0.6 eV is depicted in Figure 4b. Analogous to observations on the (001) plane, the bands are found to be degenerate at the bulk *Γ* point (*hν* = 100 eV, *k_z_* = 6 π/*a*′), while split at non‐high‐symmetry momenta (*hν* = 82 eV, *k_z_* = 5.5 π/*a*′), as illustrated in Figure 4c. The magnitude of SS parallel to $\bar{E}$‐$\bar{D}$ is measured to be also around 0.8 eV (green dashed line in Figure 4c). Although DFT calculations predict an even larger SS of about 1.1 eV, the “spin‐up” band (red) close to *E*_F_ is not seen in our ARPES data.

In an ideal scenario, similar to MnTe_2_,^[^ ^30^ ^]^ utilizing spin‐resolved ARPES to directly visualize the spin polarization texture would offer the most intuitive demonstration of the unique spin properties in CrSb. However, possible formation of magnetic domains, each much smaller than the size of the beam spot, presents challenges in obtaining reliable spin‐resolved spectroscopic data. Nonetheless, it is still possible to distinguish the spin structure of SS antiferromagnets from other signatures of the bands using spin‐integrated ARPES. First, the bands of CrSb do not split at high symmetry points, which rules out the possibility of Zeeman SS. Subsequently, since CrSb crystals are centrosymmetric, the bulk Dresselhaus and Weyl SS are prohibited.^[^ ^42^, ^43^, ^44^, ^45^, ^46^, ^47^, ^48^, ^49^, ^50^ ^]^ The surface Rashba effect, whose SS has the same magnitude along the out‐of‐plane direction, contradicts our experimental findings shown in Figure 2 that SS becomes zero at the high symmetry planes. According to our calculational results, the sign of spin polarization reverses with a reversal of *k_z_*, and the size of the SS varies along both the in‐plane and out‐of‐plane directions. This signifies a spin texture different from the classical Rashba and Dresselhaus SS which are governed by linear terms of *k*.^[^ ^51^, ^52^ ^]^ Our ARPES data proves the latter of the two characteristics, that SS exists at non‐high‐symmetry *k_z_*’s but has nodes at the bulk *ΓMK* plane. Thus, we conclude that our data reveals a new type of SS induced by unconventional antiferromagnetism.

## 3 Summary and Outlook

In summary, we have successfully conducted a systematic study of the 3D anisotropic SS band structure of CrSb using ARPES measurements and DFT calculations. The SS of CrSb shows clear *k_z_* dependence and reveals significant anisotropy along in‐plane directions. The ARPES‐measured maximum energy separation of the split bands is no less than 0.8 eV. Such electronic structure matches our theoretical results, yielding direct, high‐resolution spectroscopic evidence for the existence of significant AFM‐induced SS in unconventional antiferromagnet CrSb.

Compared to the much‐studied semiconducting candidate MnTe, CrSb is a metal with a Néel temperature much higher than the room temperature (≈703 K), making it more favorable for devices that are functional at room temperature. Moreover, CrSb exhibits a greater SS strength than what is reported in MnTe (≈0.37 eV),^[^ ^36^ ^]^ indicating that it is more easily controllable by electric fields. Recently, the 100% field‐free switching of the Néel vector was observed in CrSb.^[^ ^38^ ^]^ This demonstrates the great potential of using CrSb to design magnetic‐field‐free high‐density memory devices. Consequently, our work establishes a firm basis for further studies on potential spintronic applications based on the unique properties of CrSb.

## 4 Experimental Section

### Sample Growth and XRD Characterization

Single crystals of CrSb with (00*l*) cleavage plane were grown using the chemical vapor transport method. Starting elements (Cr powder from Aladdin, 99.5% purity; Te powder from Aladdin, 99.5% purity; I_2_ from Aladdin, 99.9% purity) were grounded and mixed thoroughly in the agate mortar with a molar ratio of Cr:Sb:I_2_ = 1:1:0.1. The mixture was then sealed into a silica tube under vacuum. The sealed ampoule was heated in a two‐zone furnace to a low‐temperature *T*_L_ = 750 °C and a high‐temperature *T*_H_ = 850 °C in 12 h and maintained at this condition for two weeks. Millimeter‐sized hexagonal‐shaped CrSb single crystals were then obtained. Single crystals of CrSb with (*l*00) cleavage plane were grown using the self‐flux method. Starting elements (Cr plates from Aladdin, 99.5% purity; Te ingots from Aladdin, 99.5% purity) were packed into an alumina crucible with a molar ratio of Cr:Sb = 3:7. The crucible was then sealed in a quartz ampoule under vacuum. The sealed ampoule was heated for 6 h up to 1000 °C, held for 20 h, then slowly cooled down to 750 °C over 100 h, at which temperature the excess Sb‐flux was removed by centrifugation. Millimeter‐sized needle‐like CrSb single crystals were then obtained (Figure S1a, Supporting Information). The CrSb samples were characterized by XRD at room temperature using a Rigaku SmartLab diffractometer with Cu Kα radiation. The diffraction pattern in Figure 1f confirms that the cleaving planes of the first type of crystals are parallel to the (00*l*) crystallographic orientation. The XRD result of CrSb samples with (l00) cleavage plane is shown in Figure S1 (Supporting Information).

### Energy Dispersive X‐Ray Spectroscopy Measurement

EDX experiment was operated on Nova NanoSem450 at an accelerating voltage of 20 kV and a current of 1 nA.

### Laue X‐ray Diffraction Measurement

Laue X‐ray diffraction measurement was performed on a Laue crystal orientation system (LCS2020W) designed by the Shanghai Institute of Ceramics, Chinese Academy of Sciences. During the measurement, the sample was positioned at a distance of 5 cm from the X‐ray source. The exposure time was set to 120 s to ensure an adequate signal‐to‐noise ratio.

### Magnetic Characterization

Magnetization measurements of the CrSb single crystals were carried out with a superconducting quantum interference device (SQUID)‐vibrating sample magnetometer (VSM) system (MPMS3, Quantum design). This system is capable of cooling samples down to 1.8 K and can generate a variable magnetic field up to ±7 T along both in‐plane and out‐of‐plane directions. FC and ZFC curves were measured by increasing the temperature from 2 to 400 K with both in‐plane and out‐of‐plane magnetic fields of 500 Oe.

### ARPES Measurements

The *k_z_* dispersion data and the spin‐integrated electronic structure at different photon energies were performed at BL03U^[^ ^53^ ^]^ and BL09U of the Shanghai Synchrotron Radiation Facility (SSRF), BL09A of the Hiroshima Synchrotron Radiation Center (HiSOR), and BL25SU of the SPring‐8 synchrotron facility. Data at BL03U of the SSRF were measured with a Scienta Omicron DA30 electron analyzer and *p*‐polarized light with photon energies between 40 and 120 eV. Data at BL09U of the SSRF were measured with a Scienta Omicron DA30 electron analyzer and *p*‐polarized light with photon energies between 94 and 144 eV. Data at BL09A of HiSOR were measured with a SPECS ASTRAIOS 190 electron detector with wide detector angle and *p*‐polarized light with photon energies between 11 and 40 eV. Data at BL25SU of SPring‐8 were measured with a Scienta Omicron DA30 electron analyzer and *C^+^*‐polarized light with photon energies between 400 and 650 eV. The measurement temperature was set to around 30 K at SSRF and HiSOR and around 77 K at SPring‐8. The samples for all ARPES measurements were cleaved in situ and measured in a vacuum better than 2 × 10^−10^ Torr.

### First‐Principles Calculations

The electronic structure calculations were carried out using the DFT method encoded in the Vienna Ab‐initio Simulation Package (VASP)^[^ ^54^, ^55^ ^]^ based on the projector augmented wave (PAW) method.^[^ ^56^ ^]^ The Perdew–Burke–Ernzerhof (PBE) approximation was used for the exchange‐correlation function.^[^ ^57^ ^]^ The plane‐wave cutoff energy was set to 520 eV. GGA+*U* correction was applied to the Cr 3*d* orbitals, and *U* was set to be 1.0 eV. The *k*‐point sampling is 10 × 10 × 6 with the *Γ* scheme for the bulk structure. To study the CECs of CrSb along (001), maximally localized Wannier functions were determined using a reduced basis set formed by the *d* orbitals of Cr, *s*, and *p* orbitals of Sb atoms in the Wannier90 software.^[^ ^58^ ^]^ The WannierTools package was used to simulate the theoretical CECs.^[^ ^59^ ^]^ The experimental values used for cell parameters were *a* = *b* = 4.18 Å, and *c* = 5.46 Å. Atomic positions were fully relaxed until the force on each atom was smaller than 1 × 10^−3^ eV Å^−1^, and the total energy convergence criterion was set to be 1 × 10^−7^ eV.

### Statistical Analysis

The intensity in the ARPES *E‐k* cuts in Figures 2c and 3c was normalized using the area normalization method. After normalization, symmetrization of the intensity was performed with respect to the high symmetry line *k_x_* = 0.

## Conflict of Interest

The authors declare no conflict of interest.

## Supporting information

Supporting Information

## Data Availability

The data that support the findings of this study are available on request from the corresponding author. The data are not publicly available due to privacy or ethical restrictions.
