# Supplementary material for: Observation of Spin Splitting in Room‐Temperature Metallic Antiferromagnet CrSb
Source: Adv Sci (Weinh). 2024 Sep 20;11(43):2406529. doi: 10.1002/advs.202406529 (PMC11578308; doi:10.1002/advs.202406529)
Supplement: Supplementary file 1 — Supporting Information [file ADVS-11-2406529-s001.pdf]

ADVANCED SCIENCE
Open Access

Supporting Information

for *Adv. Sci.*, DOI 10.1002/advs.202406529

Observation of Spin Splitting in Room-Temperature Metallic Antiferromagnet CrSb

*Meng Zeng, Ming-Yuan Zhu, Yu-Peng Zhu, Xiang-Rui Liu, Xiao-Ming Ma, Yu-Jie Hao, Pengfei Liu, Gexing Qu, Yichen Yang, Zhicheng Jiang, Kohei Yamagami, Masashi Arita, Xiaoqian Zhang, Tian-Hao Shao, Yue Dai, Kenya Shimada, Zhengtai Liu, Mao Ye, Yaobo Huang, Qihang Liu and Chang Liu**

Supporting Information

Observation of Spin Splitting in Room-Temperature Metallic Antiferromagnet CrSb

Meng Zeng[1]*, Ming-Yuan Zhu[1]*, Yu-Peng Zhu[1]*, Xiang-Rui Liu[1], Xiao-Ming Ma[1], Yu-Jie Hao[1], Pengfei Liu[1], Gexing Qu[2], Yichen Yang[3], Zhicheng Jiang[4], Kohei Yamagami[5], Masashi Arita[6], Xiaoqian Zhang[8], Tian-Hao Shao[1], Yue Dai[1], Kenya Shimada[6], Zhengtai Liu[3], Mao Ye[3], Yaobo Huang[7], Qihang Liu[1], Chang Liu[1]†

[1]*Department of Physics and Shenzhen Institute for Quantum Science and Engineering (SIQSE), Southern University of Science and Technology (SUSTech), Shenzhen, Guangdong 518055, China*
[2]*Beijing National Laboratory for Condensed Matter Physics and Institute of Physics, Chinese Academy of Sciences, Beijing 100190, China*
[3]*State Key Laboratory of Functional Materials for Informatics, Shanghai Institute of Microsystem and Information Technology, Chinese Academy of Sciences, Shanghai 200050, China*
[4]*National Synchrotron Radiation Laboratory and School of Nuclear Science and Technology, University of Science and Technology of China, Hefei, Anhui 230026, China*
[5] *Japan Synchrotron Radiation Research Institute (JASRI), Sayo, Hyogo 679-5198, Japan*
[6]*Hiroshima Synchrotron Radiation Centre, Hiroshima University, Higashi-Hiroshima, Hiroshima 739-0046, Japan*
[7]*Shanghai Synchrotron Radiation Facility, Shanghai Advanced Research Institute, Chinese Academy of Sciences, Shanghai 201204, China*
[8]*Key Laboratory of Quantum Materials and Devices of Ministry of Education, School of Physics, Southeast University, Nanjing Jiangsu 211189, China*

*These authors contribute equally to this work.
†Corresponding author. E-mail: liuc@sustech.edu.cn

Keywords: unconventional antiferromagnet, spin splitting antiferromagnet, spintronics, angle-resolved photoemission spectroscopy, density functional theory calculations

I. XRD characterization of CrSb crystals cleaved along the (100) plane

The CrSb samples with the (100) cleavage plane were characterized by XRD at room temperature using a Rigaku SmartLab diffractometer with Cu Kα radiation. The diffraction pattern in Figure S1b) resolves characteristic diffraction peaks of CrSb without signals from impurities, proving the high quality of the single crystals.

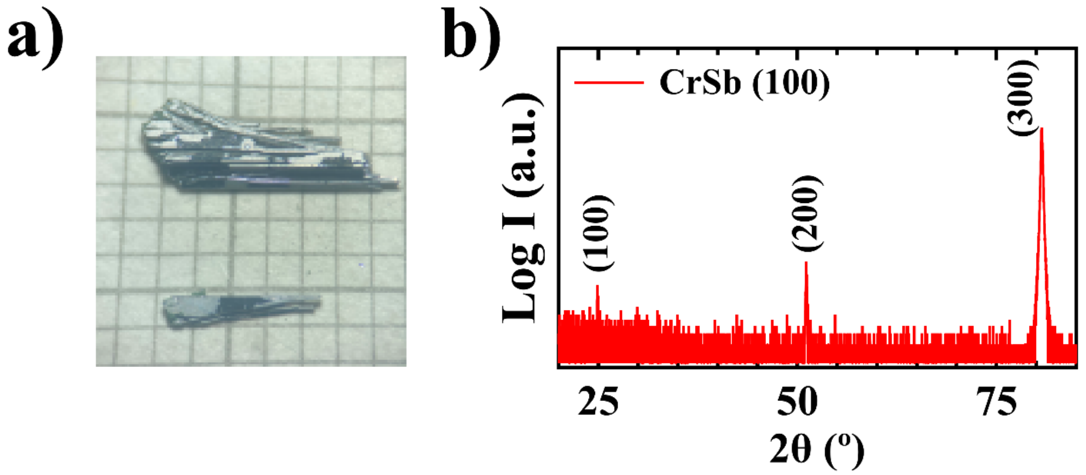

Figure S1. Phase characterization of CrSb single crystals with the (100) plane exposed. a) Typical CrSb single crystals with (100) plane exposed. Image is taken against a millimeter grid. b) Single crystal XRD result of the (100) surface of CrSb.

II. Elemental analysis of CrSb single crystals

To confirm the uniformity of elemental distribution in the stoichiometric crystals, we conduct surface morphology mapping on the CrSb samples by scanning electron microscope (SEM). Figure S2a) demonstrates the overall flatness of the sample surface. We select seven regions marked in Figure S2a) for elemental analysis. The data are depicted in Figure S2b). A ratio close to Cr : Sb = 1 : 1 is found in all regions, indicating a high quality of the crystals. The Sb content is found to be slightly below 50%, suggesting that there is a small amount of Sb vacancies within the sample, which explains the *p*-doped electronic structure found in our ARPES data. The elemental maps of Cr and Sb atoms within the sample are illustrated in Figure S2c)-d), indicating a homogeneous distribution of the Cr and Sb elements.

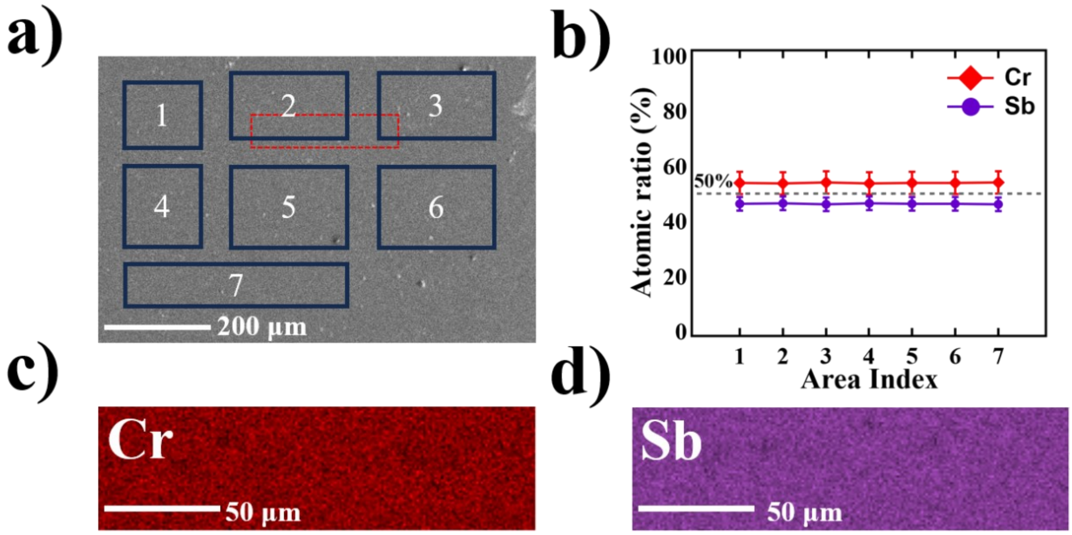

Figure S2. Elemental analysis of CrSb single crystals. a) Surface morphology of the CrSb single crystals. The blue/red boxes represent the integration areas for the energy-dispersive X-ray spectroscopy (EDS) data in Penal b) / Penal c)-d). b) Atomic ratio of Cr and Sb at the seven regions indexed in (a). c)-d), EDS elemental maps of Cr and Sb atoms.

III. Magnetic properties of CrSb single crystals

We characterize the magnetic properties of CrSb using the magnetic properties measurement system (MPMS), as shown in Figure S3. Field cool (FC) and zero field cool (ZFC) temperature dependence of magnetization are measured from 2 to 400 K with a small magnetic field of $H = 500$ Oe applied along both in-plane and out-of-plane directions, as depicted in Figure S3a). Since the Néel temperature of CrSb (~703 K) is significantly higher than the temperature range of our measurement, this figure cannot reach the magnetic phase transition point, so the FC and ZFC moment vs. temperature (*M-T*) curves overlap. The magnetic moment decreases gradually as temperature decreases until $T < 10$ K, where the curves experience a slight upturn. Figure S3b) shows the field dependence of magnetization at different temperatures, with a maximum field $H//c = \pm 2$ T at 2 K and ±7 T at other temperatures. It can be observed that the curves for all temperatures are linear, passing through the origin of the coordinates. Combined with the *M-T* curves, it can be inferred that CrSb is in an antiferromagnetic ground state within 2 - 400 K.

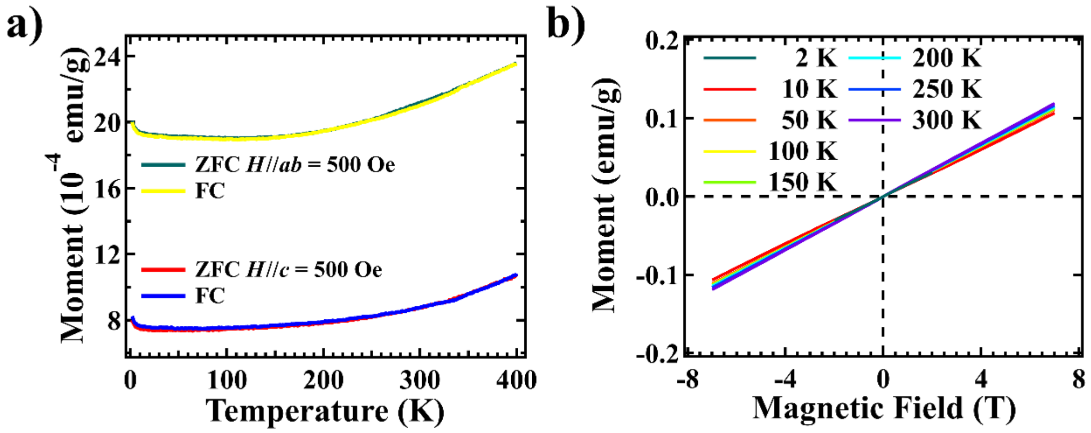

Figure S3. Temperature and magnetic field dependence of the magnetic moment. a) FC and ZFC temperature dependence of moment with *H//c* and *H//ab* = 500 Oe. b) Magnetic field dependence of the magnetic moment at different temperatures.

IV. Computational evidence for the non-relativistic spin splitting induced by unconventional antiferromagnetism

According to the symmetry analysis mentioned in the main text, CrSb is one of the spin-split (SS) antiferromagnets whose band structure experiences a substantial lift of the Kramers degeneracy at non-high-symmetry momenta. The resultant SS is non-relativistic and insensitive to the SOC strength. To verify this in first-principles calculations, in Figure S4 we compared DFT-calculated bulk bands of CrSb for three scenarios: a non-magnetic ground state with SOC (left panel) and an antiferromagnetic ground state with or without SOC (middle and right panels). It is seen that the bulk bands are spin degenerated for all *k*-points in the nonmagnetic case, which is markedly different from the significant band splitting observed in Figures 2-4 in the main text. Comparing the calculated band structure of the antiferromagnetic ground state with and without SOC, we notice that the energy scale of SS is nearly unchanged no matter whether SOC is considered or not. The existence of SOC only slightly lifts the band degeneracy at the high symmetry points. Therefore, the substantial SS in CrSb is induced overwhelmingly by the antiferromagnetic order, SOC gives only a secondary effect to the electronic structure.

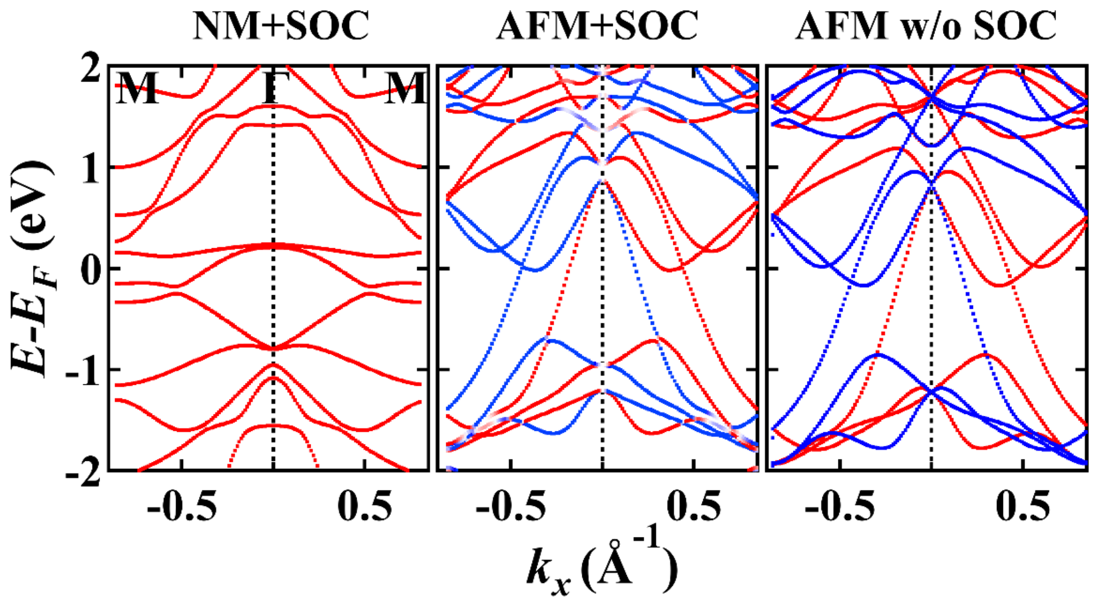

Figure S4. Computational evidence for the non-relativistic SS induced by unconventional antiferromagnetism. The three panels show respectively the DFT bulk band structure along $\bar{\Gamma}$-$\bar{M}$-$\bar{\Gamma}$ calculated under (left) a non-magnetic (NM) ground state with SOC considered, (middle) an antiferromagnetic (AFM) ground state with SOC and (right) without SOC.

V. Comparison of experimental data and calculational results at different k_z's

Using the data given in Figure 2 of the main text as well as Figure S5 below, we have determined the inner potential V_0 to be 12.8 eV in the formula $k_z = \sqrt{2m/\hbar^2(E_k \cos^2\theta + V_0)}$ by comparing the experimental and the calculational results. Using this V_0, the ARPES-measured photon energies corresponding to the bulk Γ points ($k_z = 2n\pi$, $n = 0, 1, 2, 3, \ldots$) are 72 eV, 118 eV, 495 eV, and 600 eV, respectively.

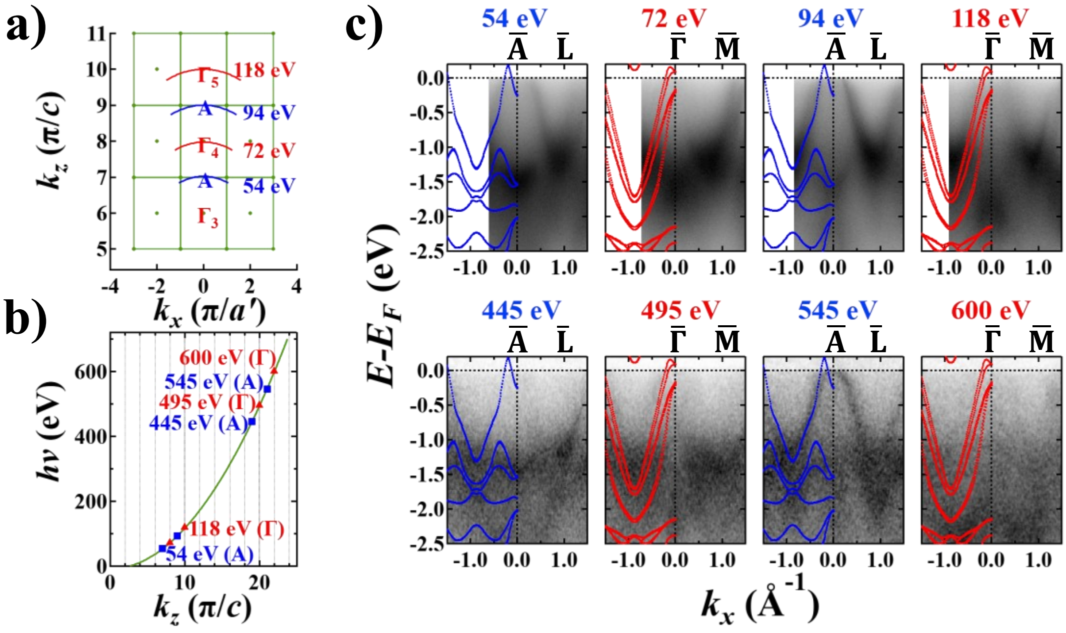

Figure S5. Relationship between $h\nu$ and k_z. a) Cross section of the Brillouin zone along the $k_{[001]}$-$k_{[110]}$ plane (k_z-k_x plane in the figure). b) The relationship between $h\nu$ and k_z for CrSb obtained from the $h\nu$-dependent data. Blue squares/red triangles mark the photon energies corresponding to bulk Γ/A points. c) ARPES band dispersion parallel to $\bar{M}$-$\bar{\Gamma}$-$\bar{M}$ at different photon energies marked in b), compared with DFT calculational results (the appended blue/red lines).

VI. Comparison of experimental data and calculational results at different k_z's for samples cleaved along the (100) plane

Using the data given in Figure 4b) of the main text, we have determined the inner potential V_0 to be 11.8 eV for the samples cleaved along the (100) plane. Using this V_0, the ARPES-measured photon energy corresponding to the bulk Γ point is 100 eV, whereas those corresponding to the bulk M points are 66 eV and 137 eV [Figure S6b)]. Similar to the samples cleaved along the (001) plane, the E-k maps of the corresponding photon energies agree well with the calculation results, indicating the accuracy of V_0.

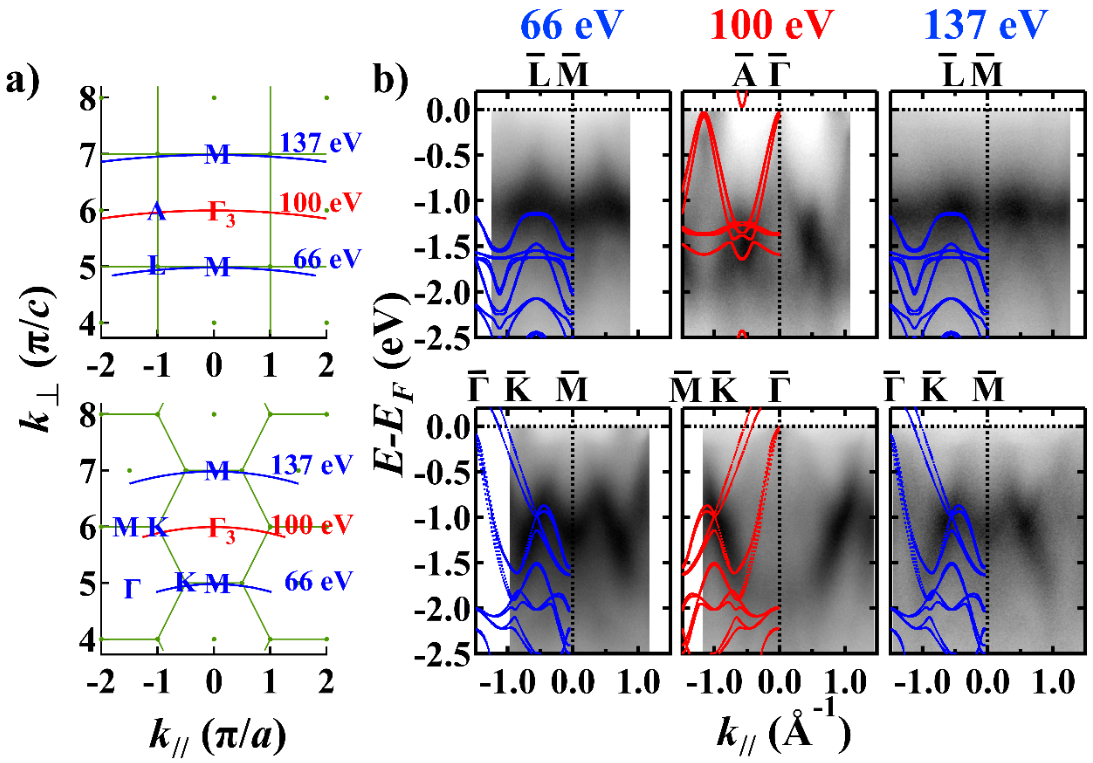

Figure S6. Relationship between $h\nu$ and k_z for the samples cleaved along the (100) plane. a) Cross section of the Brillouin zone along the ΓAML plane (top) and ΓKM plane (bottom). b) ARPES band dispersions parallel to $\bar{A}$-$\bar{\Gamma}$-$\bar{A}$ and $\bar{M}$-$\bar{K}$-$\bar{\Gamma}$-$\bar{K}$-$\bar{M}$ at photon energies marked in a), compared with DFT-calculated results (the appended blue/red lines).

VII. Detailed analysis of the Sb peaks in the XPS curve

We enlarged the region of E_B = 26 - 38 eV in Figure 1e), and performed peak fitting on the curve, as shown in Figure S7 below. Four peaks were recognized after fitting, which are divided into two groups and marked in pink and purple colors. The difference between the two peaks within each group is 1.2 eV. By comparing with the data from the NIST XPS database, we found that the two purple peaks correspond to the $4d_{3/2}$ and $4d_{5/2}$ orbitals of elemental Sb, while the two pink peaks correspond to the $4d_{3/2}$ and $4d_{5/2}$ orbitals of Sb^{3+} ions. The appearance of elemental Sb in the curve may be due to the presence of a small amount of unreacted elemental Sb on the sample surface.

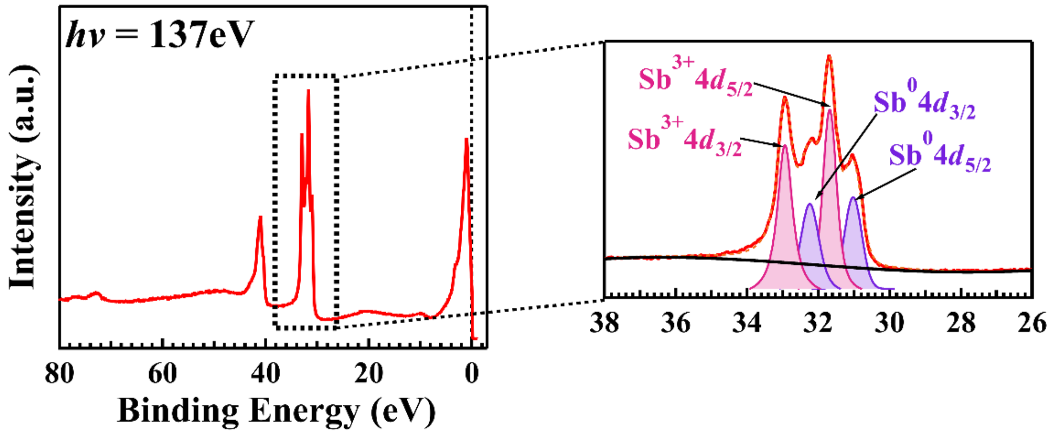

Figure S7. Detailed analysis of the Sb peaks in the XPS curve. The binding energy region between 26 eV and 38 eV is enlarged and shown in the right panel. Four peaks have been identified after Lorentzian curve fitting. The two purple peaks correspond to the $4d_{3/2}$ and $4d_{5/2}$ orbitals of elemental Sb, while the two pink peaks correspond to the $4d_{3/2}$ and $4d_{5/2}$ orbitals of Sb^{3+} ions. The appearance of elemental Sb in the curve may be due to the presence of a small amount of unreacted elemental Sb on the sample surface.

VIII. Evolution of band splitting at different out-of-plane momentum positions under soft-X-ray incident photons

The E_B-k_x dispersion of CrSb at different k_z's in soft-X-ray energy region are shown in Figure S8. The cuts from left to right correspond to $h\nu = 495$ eV ($k_z = 20\ \pi/c$), 510 eV ($k_z = 20.3\ \pi/c$), 520 eV ($k_z = 20.5\ \pi/c$), 530 eV ($k_z = 20.7\ \pi/c$) and 545 eV ($k_z = 21\ \pi/c$), respectively. The energy scale of the splitting varies with photon energies in a similar sense as the behavior shown in Figure 2c of the main text: no splitting is seen at $h\nu = 495$ and 545 eV (the bulk GMK and AHL planes), while the maximum of band splitting is (roughly) seen at $h\nu = 520$ eV (the middle plane between GMK and AHL).

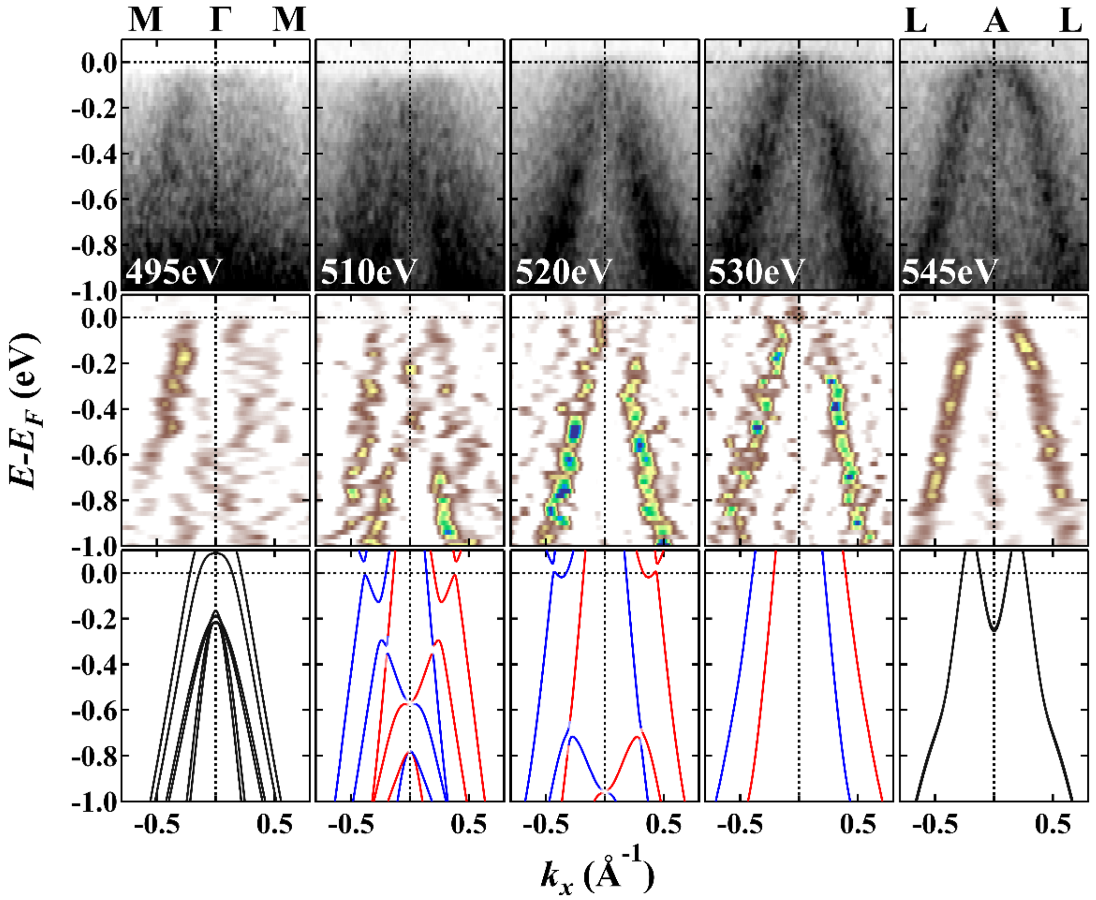

Figure S8. Evolution of band splitting at different out-of-plane momentum positions under soft-X-ray incident photons. From top to bottom: ARPES band dispersion parallel to $\bar{\Gamma}$-$\bar{M}$ at different photon energies; corresponding 2nd derivative analysis along the EDCs; DFT-calculated bulk bands, where red and blue correspond to out-of-plane spin-up and -down polarization, respectively. All calculated bands are shifted up by 0.15 eV to account for the charged defects in real crystals.

IX. Detail analysis of the ARPES *E-k* cuts taken with low incident photon energies

To explain why we decided to choose the photon energies from 94 to 118 eV for data analysis in the main text, we analyzed the energy bands in the low photon energy range (12 – 37 eV) and summarized the results in Figure S9. The central message in Figure S9 is that the bands observed at the low photon energy range most likely originate from surface bands rather than bulk states, while signal from surface bands is much weaker than that from the bulk states for $94 < h\nu < 118$ eV. To see that, we

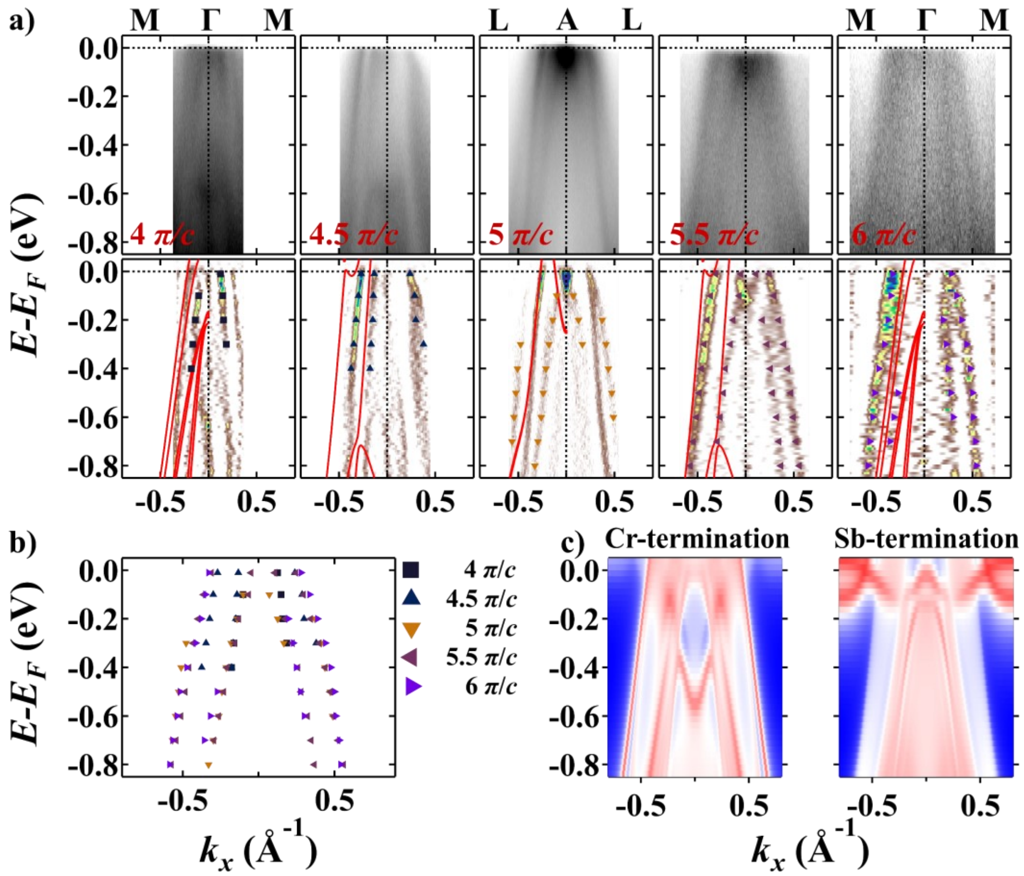

Figure S9. Detail analysis of the ARPES *E-k* cuts taken with low incident photon energies. a) First row: ARPES band dispersion parallel to $\bar{\Gamma}$-$\bar{M}$ taken at different photon energies (corresponding k_z values are marked in red). Second row: corresponding second derivative analysis along the EDCs. Different types of marks are used for locating the band positions. b) Overlay of band positions taken under different photon energies. c) Calculational results of the surface states for the two different terminations. Red color represents the surface states, while blue color represents the background.

extracted the positions of the most resolvable bands on each raw *E-k* maps under 12 < $h\nu$ < 37 eV photons in the first row of Figure S9a), and overlaying them on a single panel [Figure S9b)]. We found that the band positions do not vary with k_z values, implying that they are likely surface states. Moreover, positions of the bands in Figure S9b) deviate significantly from those of the bulk bands, which are plotted as red curves in the second row of Figure S9a) (2nd derivative maps of the first row). To further elucidate the origin of these bands, we used the surface Green's function (SGF) method to calculate the surface states. In the calculation, we constructed the SGF from the bulk Hamiltonian by the WANNIER90 package. Before constructing the Hamiltonian, we slightly shifted the atoms from the edge of the unit cell to avoid dangling bonds. The calculated results are shown in Figure S9c). In the figure, red color represents the surface states, while blue color represents the background. As observed, in the Cr-terminated case, two sharp linear bands appear on either side of the Γ point, and their positions match well with the bands shown in Figure S9b). Therefore, these bands most likely originate from the surface states. Signal from the surface states is particularly strong at low photon energies, possibly due to an enhanced ARPES matrix element.

X. Comparison of ARPES data and calculated band structures using different *U* values

During the data analysis, we calculated the relaxed lattice parameters and the band structures using different *U* values and compared them with the experiment. When $U = 1$ eV, the calculated lattice parameters are consistent with the experimental ones. This is the main reason we used this value of *U*. Figure S10 shows a comparison of band structure between the ARPES experiment and the calculated ones using different *U* values. Table S1 shows a comparison of lattice parameters between the experiment and the calculated ones under different *U* values.

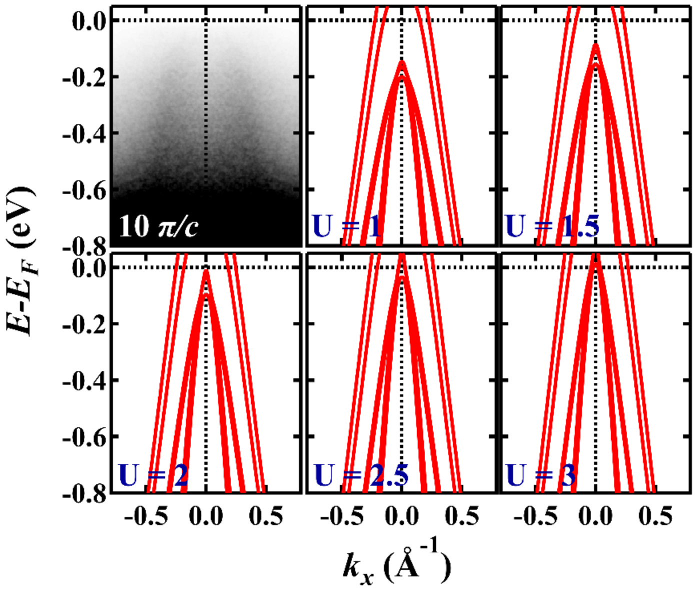

Figure S10. Comparison of ARPES data and calculated band structures parallel to $\bar{\Gamma}$-$\bar{M}$, using different *U* values.

Table S1. Relaxed lattice parameters under different *U* values

U	*a* (Å)	*c* (Å)
0	4.143	5.271
0.5	4.154	5.346
1	4.165	5.427
1.5	4.184	5.493
2	4.149	5.725
2.5	4.214	5.594
3	4.23	5.631
Experiment	4.18	5.46
